# Dosimetric impact of bolus thickness and immobilization mask use in postmastectomy radiotherapy: a chest wall thickness–based analysis

**DOI:** 10.3389/fonc.2025.1716042

**Published:** 2026-01-20

**Authors:** Qing-Jun Shang, Meng Zhang, Bing-Xin Zhao, Qi Wang, Wen Gao, Bi-Yuan Zhang, Hai-Ji Wang, Tian-Hui Guo

**Affiliations:** Department of Radiation Oncology, The Affiliated Hospital of Qingdao University, Qingdao, Shandong, China

**Keywords:** breast cancer, postmastectomy radiotherapy (PMRT), chest wall thickness, compensator thickness, thermoplastic mask, dosimetric parameters

## Abstract

**Purpose:**

Postmastectomy radiotherapy (PMRT) requires a balance between optimal target coverage and organ-at-risk (OAR) sparing. Thermoplastic masks and different bolus thicknesses are frequently applied in clinical practice, yet their combined dosimetric effects and dependence on chest wall thickness remain insufficiently defined.

**Methods and materials:**

Seventeen breast cancer patients treated with PMRT were retrospectively analyzed. For each patient, six intensity-modulated radiotherapy (IMRT) plans were generated, combining the presence or absence of a thermoplastic mask with bolus thicknesses of 0, 3, and 5 mm. Dosimetric parameters of the skin, planning target volume (PTV), OARs, and treatment efficiency (monitor units, MU) were compared across plans. Subgroup analyses were performed according to chest wall thickness (<4.1 cm, 4.1–5.0 cm, >5.0 cm). Correlations between chest wall thickness and dosimetric indices were evaluated using Spearman’s rank analysis.

**Results:**

Application of bolus improved dose homogeneity and PTV coverage, reduced lung dose and MU, but increased skin dose. The thermoplastic mask alone raised skin dose, functioning as an unintended compensator. In patients without masks, bolus effects on skin and OARs were more pronounced, suggesting greater sensitivity to compensation thickness. Chest wall thickness demonstrated negative correlations with skin dose, lung exposure, and MU. Patients with thin chest walls (<4.1 cm) derived the greatest benefit from bolus application, whereas patients with thick chest walls (>5.0 cm) showed minimal dosimetric improvement.

**Conclusions:**

Both thermoplastic masks and bolus significantly affect PMRT dosimetry. Bolus provides improved PTV coverage and lung sparing at the cost of higher skin dose, while thermoplastic masks act as unintended compensators. Chest wall thickness strongly influences these effects, supporting individualized selection of bolus and mask use, particularly for patients with thinner chest walls.

## 1 Introduction

Breast cancer is the most common malignancy in women; despite improved survival, treatment optimization remains challenging (1, 2).

The standard treatment for breast cancer often involves a combination of surgery, systemic therapy (chemotherapy, hormonal therapy, and targeted therapy), and radiotherapy (RT) (3). Post-mastectomy radiotherapy (PMRT) is essential for reducing local recurrence and improving survival, especially in high-risk patients (4, 5). Technical factors such as immobilization devices and bolus application can influence target delineation and dose distribution (6, 7), yet the specific effects of different mask applications and compensation thicknesses on radiotherapy planning remain insufficiently studied.

Dosimetric evaluation in PMRT reflects the precision and uniformity of dose distribution. This study assesses the effects of thermoplastic mask use and compensation thickness on PMRT dosimetry, focusing on skin dose, target coverage, organ-at-risk exposure, and treatment efficiency. The influence of chest wall thickness on these parameters is also analyzed to clarify its role in target coverage and normal tissue sparing.

## 2 Materials and methods

### 2.1 Clinical data

Adhering to stratified design principles—specifically methodological guidelines for stratified dosimetric studies and non-parametric test requirements that recommend ≥5 subjects per subgroup to avoid statistical inefficiency and ensure valid intragroup comparisons via the Friedman test (8) -and building on numerous high-quality radiation oncology dosimetric studies (especially those with stratified designs analogous to ours) (9, 10), we ultimately enrolled 17 breast cancer patients who underwent modified radical mastectomy and required postoperative radiotherapy at The Affiliated Hospital of Qingdao University (June–December 2024, 10 left-sided, 7 right-sided). These patients were stratified by average chest wall thickness into three subgroups: low (<4.1 cm, n=6), intermediate (4.1–5.0 cm, n=5), and high (>5.0 cm, n=6), with each subgroup comprising at least 5 patients to fulfill the purpose of our stratified research. This study was approved by the ethics committees of the Affiliated Hospital of Qingdao University (Reference No. QYFYWZLL30486). CT simulation was performed with 3-mm slices from mandible to the second lumbar vertebrae.

### 2.2 Target area and organ at risk sketching

For each patient, two imaging sets were created with and without a thermoplastic mask to assess immobilization and bolus effects. Image registration was performed for alignment. The clinical target volume (CTV) included the chest wall and regional lymphatic areas, and a single oncologist ensured consistency. The PTV was generated by adding a 5-mm margin to the CTV with a 2-mm subcutaneous retraction. Skin dose was assessed using 3- and 5-mm sub-PTV layers. OARs (lungs, heart, spinal cord, contralateral breast, thyroid) were delineated.

### 2.3 Plan design

Treatment planning was conducted using the Varian Eclipse v15.6 treatment planning system (TPS) and delivered via a Varian Trilogy linear accelerator equipped with a 60-leaf multiple leaf collimator (MLC). For each patient, a total of six fixed-jaw intensity-modulated radiotherapy (fix jaw-IMRT) plans were created, divided into two groups by bolus application status (with/without), and each group comprised 3 subgroups stratified by bolus thickness (**Table 1**):

**Table 1:** Radiotherapy plan grouping according to thermoplastic mask use and bolus thickness.

	Group 1(P-mask)	Group 2(P-nomask)
Subgroup 1	0mm bolus (P-mask)	0mm bolus (P-nomask)
Subgroup 2	3mm bolus (P-mask-0.3)	3mm bolus (P-nomask-0.3)
Subgroup 3	5mm bolus (P-mask-0.5)	5mm bolus (P-nomask-0.5)

*Virtual water-equivalent material with an electron density of 1.0.

All treatment plans, which were designed by the same senior medical physicist, used 6MV X-rays, a fixed dose rate of 400 MU/min, and the anisotropic analytical algorithm (AAA) for dose calculation with a default grid resolution of 2.5 mm. The prescribed dose was 50 Gy in 25 fractions (5000 cGy/25F), ensuring 95% coverage of the target volume by the isodose line for normalization.

### 2.4 Plan evaluation

Plans were analyzed for four dosimetric categories, and chest wall thickness was correlated with dose distribution using dose–volume histograms (DVH).

#### 2.4.1 Dosimetric parameters of the skin structure

The skin was defined at 3 and 5 mm below the surface to reduce surface dose uncertainty, consistent with prior studies (11, 12). Parameters included D_max_, D_min_, D_mean_, and skin volumes receiving ≥105% (V_105%_) and ≥110% (V_110%_) of the prescription dose.

#### 2.4.2 Dosimetric parameters of the target volume

Parameters included:

1. Homogeneity index (HI), defined as (13):


$$HI\;=\;\left(D_{2}-\;D_{98}\right)/D_{50,}$$

where D_2_, D_98_ , and D_50_ represent the minimum doses covering 2%, 98%, and 50% of the target volume, respectively. A lower HI value, approaching zero, indicates better homogeneity of the target dose distribution.

2. Conformity index (CI), defined as (14):


$$\text{CI }=\;\left({\text{V}}_{\text{T},\text{ ref}}/{\text{V}}_{\text{T}}\right)\;\times \;\left({\text{V}}_{\text{T},\text{ ref}/}{\text{V}}_{\text{ref}}\right),$$

where V_T, ref_ is the target volume covered by the prescription dose, V_T_ is the total target volume, and V_ref_ is the total volume covered by the prescription dose. A CI value closer to 1 indicates better conformity of the target volume.

3. PTV dose parameters, including:

- D_max_: Maximum dose within the PTV.
- V_95%_: The percentage of the PTV receiving at least 95% of the prescription dose.

#### 2.4.3 Dosimetric parameters of OARs

1. Lungs:
  - V_5_, V_20_, V_40_: The percentage of lung volume receiving at least 5 Gy, 20 Gy, and 40 Gy, respectively.
  - D_mean_: Mean lung dose.
2. Heart:
  - V_5_, V_10_, V_20_: The percentage of heart volume receiving at least 5 Gy, 10 Gy, and 20 Gy, respectively.
  - D_mean_: Mean heart dose.

#### 2.4.4 Radiation delivery efficiency

Treatment efficiency was assessed by monitor units (MU); lower MU indicates higher cost-effectiveness.

#### 2.4.5 Chest wall thickness

Defined as the perpendicular distance from skin to rib surface. Measurements at upper (thoracic inlet), middle (midpoint between inlet and xiphoid), and lower (xiphoid) chest wall were averaged. Patients were grouped as:

- Low chest wall thickness: < 4.1 cm.
- Intermediate chest wall thickness: 4.1–5.0 cm.
- High chest wall thickness: > 5.0 cm.

### 2.5 Statistical analysis

All statistical analyses were performed using SPSS (v19.0, IBM Corp., Armonk, NY, USA). Continuous variables are presented as medians with interquartile ranges (IQRs). Dosimetric parameters among the six radiotherapy plans were compared using the Friedman test; significant results were followed by Bonferroni-adjusted Wilcoxon signed-rank tests.

Subgroup analyses by chest wall thickness (<4.1 cm, 4.1–5.0 cm, >5.0 cm) used the same procedures. Correlations between chest wall thickness and dosimetric parameters were assessed with Spearman’s rank coefficient. All tests were two-sided, with p < 0.05 considered significant; Bonferroni correction was applied for multiple comparisons.

## 3 Results

Comparison of the dosimetric parameters of the six radiotherapy plans for all 17 patients are listed in **Table 2**.

**Table 2:** Comparison of the dosimetric parameters of the six radiotherapy plans of patients.

Parameters	P-mask	P-mask-0.3	P-mask-0.5	P-nomask	P-nomask-0.3	P-nomask-0.5	χ2	p
Skin								
D_min_ at 3mm Depth (cGy)	3077.9(2803.75,3222.60) df	2997.20(2892.35,3352.75) df	3005.00(2864.10,3224.15) df	1363.30(1120.35,1586.90)	1940.60(1644.00,3062.75)	1891.70(1641.55,3003.75)	51.824	***
D_min_ at 5mm Depth (cGy)	3048.70(2726.40,3162.65) df	3009.70(2880.45,3331.20) df	2991.90(2759.20,3182.80) df	1395.70(1266.25,1584.65)	1867.90(1598.80,2963.25)	1844.50(1553.50,2721.10)	53.50	***
D_max_ at 3mm Depth (cGy)	5876.20(5807.85,6021.00) e	5830.20(5774.25,5911.80)	5843.00(5774.55,5936.90)	5924.90(5739.85,6597.90)	5790.20(5731.40,5860.85)	5791.70(5750.25,5881.65)	18.28	**
D_max_ at 5mm Depth (cGy)	5892.50(5833.25,6049.95) ef	5830.20(5791.40,5928.75)	5846.70(5781.05,5958.60)	5964.10(5845.10,6597.90) efb	5820.5(5782.60,5907.60)	5818.90(5772.70,5895.70)	25.30	***
D_mean_ at 3mm Depth (cGy)	5213.20(5125.95,5340.80) de	5251.70(5192.70,5314.45) de	5262.40(5201.75,5340.45) def	4327.70(4125.40,4945.35)	5054.60(4980.00,5212.95)	5141.70(5038.75,5246.65)	49.50	***
D_mean_ at 5mm Depth (cGy)	5281.40(5195.65,5364.95) de	5290.40(5230.10,5345.80) def	5288.90(5231.90,5343.70) def	4751.60(4562.25,5198.45)	5154(5063.85,5256.30)	5191.70(5100.55,5249.70)	42.69	***
Skin ≥V_105%_ at 3mm (%)	46.30(36.44,68.52) d	54.14(44.73,62.22) de	54.97(44.31,63.11) de	12.71(4.89,47.59)	31.59(22.10,54.26)	40.67(29.80,59.21)	32.50	***
Skin ≥V_105%_ at 5mm (%)	59.52(48.38,68.30) de	59.82(50.36,68.38) def	58.70(49.45,67.64) de	37.05(21.26,63.31)	42.80(33.00,61.07)	47.21(36.32,60.56)	36.80	***
Skin ≥V_110%_ at 3mm (%)	18.06(9.21,35.10) d	22.41(13.68,30.79) de	23.32(14.49,32.00) de	1.68(0.65,29.50)	8.87(3.80,18.64)	16.45(7.39,26.79)	35.52	***
Skin ≥V_110%_ at 5mm (%)	27.45(16.86,33.37) e	25.03(17.37,33.05) e	23.91(18.05,32.44) e	12.37(5.90,45.94)	15.36(8.59,24.58)	19.37(10.33,26.58)	24.73	***
Target Volume								
CI	0.85(0.83,0.87) c	0.82(0.80,0.85)	0.81(0.79,0.83) de	0.85(0.78,0.86)	0.84(0.82,0.85)	0.8226(0.80,0.84)	45.63	***
HI	0.18(0.16,0.20) f	0.17(0.16,0.18)	0.16(0.15,0.18) d	0.17(0.16,0.40) f	0.16(0.15,0.18)	0.16(0.15,0.17)	27.76	***
V_95%_ (%)	98.39(97.78,98.65) f	98.63(98.34,98.85)	98.58(98.32,98.86)	98.15(96.10,98.61) ef	98.59(98.39,98.89)	98.67(98.45,98.94)	22.48	***
D_max_(cGy)	5931.40(5831.85,6076.20)	5853.30(5811.25,5914.25) d	5858.60(5816.70,5945.30)	5947.20(5857.90,6597.90)	5878.00(5838.30,5992.35)	5864.00(5808.95,5950.2) d	19.89	***
OAR								
Ipsilateral Lung V_5_ (%)	60.38(56.71,62.97)	58.84(56.59,62.34)	59.76(57.16,62.83)	61.79(58.52,68.67) e	60.68(57.09,64.71)	60.26(57.46,64.96)	15.42	**
Ipsilateral Lung V_20_ (%)	21.51(18.24,23.30)	20.59(18.08,23.30)	20.78(18.13,23.40)	22.26(17.65,26.00)	21.09(17.57,23.64)	21.10(17.59,23.10)	10.98	ns
Ipsilateral Lung V_40_ (%)	6.07(5.34,7.13)	5.76(5.27,6.80) d	6.03(5.26,6.81)	7.32(5.49,9.42)	6.74(5.04,7.26) d	6.64(5.03,7.00) d	22.28	***
Ipsilateral Lung D_mean_ (cGy)	1204.60(1137.60,1282.45)	1181.40(1142.65,1255.10) d	1192.60(1153.60,1260.40)	1236.80(1148.65,1421.35)	1207.40(1139.45,1292.95) d	1204.10(1141.80,1258.75)	15.74	**
Heart V_5_ (%)-Left	47.63(44.85,54.98)	48.22(41.94,55.38)	48.81(45.12,56.68)	56.54(48.92,78.57)	48.01(42.18,65.85)	48.57(43.18,64.36)	13.43	*
Heart V_5_ (%)-Right	21.21(12.28,27.16)	22.83(12.13,29.46)	24.06(13.21,30.63)	22.57(17.71,41.78)	23.26(16.03,27.39)	24.02(16.52,27.89)	2.92	ns
Heart V_10_ (%)-Left	19.41(17.38,25.93)	19.93(17.11,27.00)	19.96(17.26,27.73)	23.71(19.03,42.08)	19.98(15.59,22.98)	19.70(15.75,21.72)	10.57	ns
Heart V_10_ (%)-Right	6.18(2.39,7.78)	6.36(2.23,7.40)	6.08(2.32,7.47)	6.17(3.38,9.46)	4.97(3.25,9.00)	4.89(3.24,8.92)	0.89	ns
Heart V_20_ (%)-Left	9.02(4.75,10.37)	9.01(4.74,10.81)	9.00(5.12,10.87)	10.93(7.91,12.00)	8.13(4.24,11.27)	7.63(5.02,11.31)	8.69	ns
Heart V_20_ (%)-Right	0.37(0,1.13)	0.21(0,1.20)	0.23(0.00,1.18)	0.43(0.02,1.48)	0.11(0.01,1.36)	0.10(0.01,1.32)	12.56	*
Heart D_mean_(cGy)-Left	742.50(651.55,866.50)	755.25(625.20,883.08)	758.95(647.65,898.70)	852.15(747.03,1085.15)	756.35(658.05,852.78)	762.55(667.40,817.63)	10.57	ns
Heart D_mean_(cGy)-Right	406.20(308.80,483.125)	418.00(302.85,473.70)	417.60(300.68,480.35)	446.3(301.55,530.70)	415.5(297.33,476.93)	415.95(300.45,479.45)	2.95	ns
Efficiency								
Output dose (MU)	1782.00(1653.00,1907.50)	1605.00(1536.00,1718.50) ad	1625.00(1519.05,1729.00) ad	1842.00(1637.50,2433.50)	1643.00(1501.50,1769.50) ad	1640.00(1482.00,1713.85)ad	48.59	***

A indicates a statistically significant difference compared with P-mask; b indicates a statistically significant difference compared with P-mask-0.3; c indicates a statistically significant difference compared with P-mask-0.5; d indicates a statistically significant difference compared with P-nomask; e indicates a statistically significant difference compared with P-nomask-0.3; f indicates a statistically significant difference compared with P-nomask-0.5. *p < 0.05; **p < 0.01; ***p < 0.001; ns: no statistical difference.

### 3.1 Dosimetric parameters of the skin structure

Statistically significant differences were observed among the six radiotherapy plans in terms of skin dose parameters, including D_max_, D_min_, and D_mean_ at 3mm and 5mm depths, as well as V105% and V110% (p < 0.001 for all except D_max_ at 3mm, p = 0.003).

**Figure 1:**
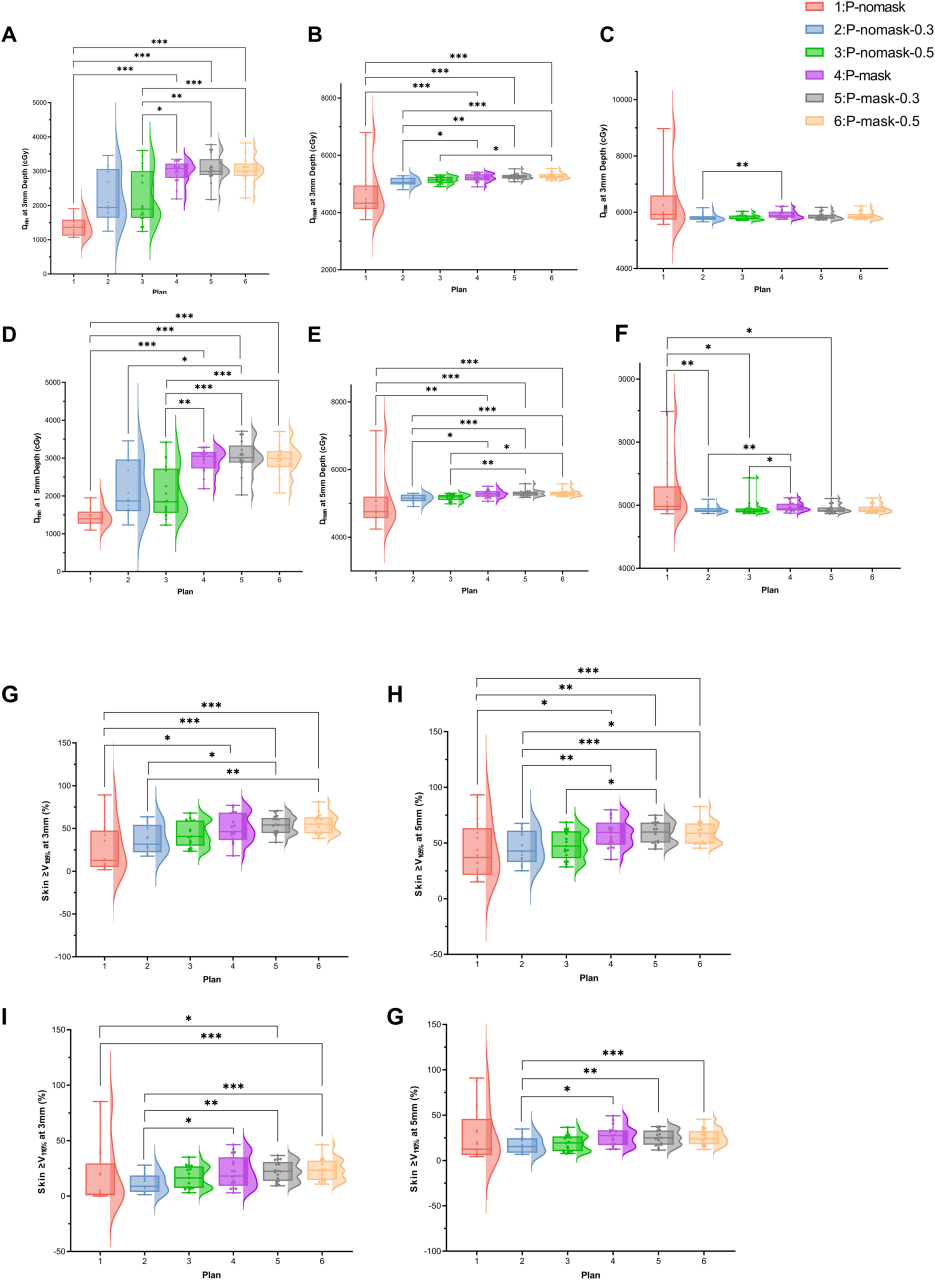
Dosimetric parameters of the skin structure. **(A)** Dmin at 3mm Depth (cGy); **(B)** Dmean at 3mm Depth (cGy); **(C)** Dmax at 3mm Depth (cGy); **(D)** Dmin at 5mm Depth (cGy); **(E)** Dmean at 5mm Depth (cGy); **(F)** Dmax at 5mm Depth (cGy); **(G)** Skin ≥V105% at 3mm (%); **(H)** Skin ≥V105% at 5mm (%); **(I)** Skin ≥V110% at 3mm (%); (J) Skin ≥V110% at 5mm (%).

From the D_min_ and D_mean_ values at 3mm and 5mm, Plan 1 (P-nomask) had the lowest skin dose. Comparison between masked and non-masked plans without bolus (Plan 4 *vs*. Plan 1) showed a significant increase in both parameters (p < 0.01), suggesting that the thermoplastic mask itself functions as a compensating layer, raising the surface dose (**Figures 1A, B, D, E**).

Since Dmax is highly variable and influenced by outliers, its clinical relevance is limited (**Figures 1C, F**).

For V105% and V110%, bolus in P-nomask plans caused a nonsignificant increase, whereas all P-mask plans showed significantly higher values than Plan 1 (p < 0.01), suggesting both mask and bolus expand high-dose regions and reduce dose (**Figures 1G–I**).

### 3.2 Dosimetric parameters of the target volume

Significant differences were observed among the six radiotherapy plans in terms of HI, CI, D_max_, and V_95%_ (p < 0.001).

#### 3.2.1 HI

**Figure 2:**
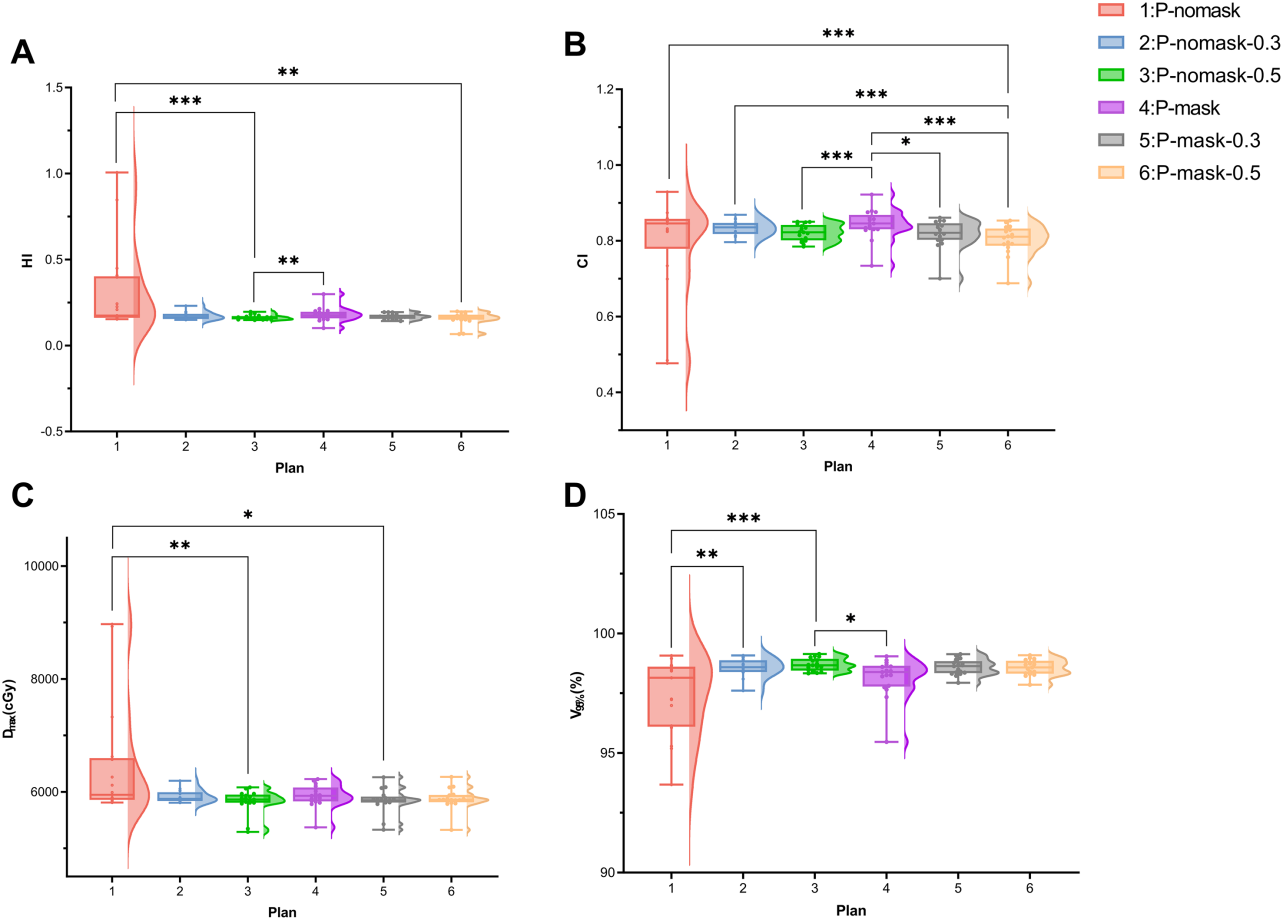
Dosimetric parameters of the target volume. **(A)** HI; **(B)** CI; **(C)** D_max_ (cGy); **(D)** V_95%_ (%). *p < 0.05, ** p < 0.01, *** p < 0.001 (Bonferroni-adjusted Wilcoxon signed-rank test following Friedman test).

Plan 3 showed significantly lower HI than Plan 1 (p < 0.001), indicating improved dose homogeneity with bolus. No significant difference was observed between Plan 1 and Plan 4, suggesting minimal impact of the thermoplastic mask (**Figure 2A**).

#### 3.2.2 CI

In the P-nomask group, Plans 2 and 3 trended toward higher CI versus Plan 1, without significance. In the P-mask group, plans 5 (p < 0.05) and 6 (p < 0.001) had significantly lower CI than Plan 4, indicating that bolus reduces conformity and increases dose heterogeneity, consistent with V105% and V110% results (**Figure 2B**).

#### 3.2.3 D_max_ and V_95%_

In the P-nomask group, bolus plans (2 and 3) showed higher V95% and lower Dmax compared to Plan 1 (Dmax: Plan 1 *vs*. 2, p < 0.01; V95%: Plan 1 *vs*. 2, p < 0.01; Plan 1 *vs*. 3, p < 0.001), indicating improved target coverage and reduced hot spots. Similar trends were observed in the P-mask group, showing bolus enhances PTV coverage and reduces dose heterogeneity in both settings (**Figures 2C, D**).

### 3.3 Dosimetric parameters of OARs

#### 3.3.1 Lung dosimetry

**Figure 3:**
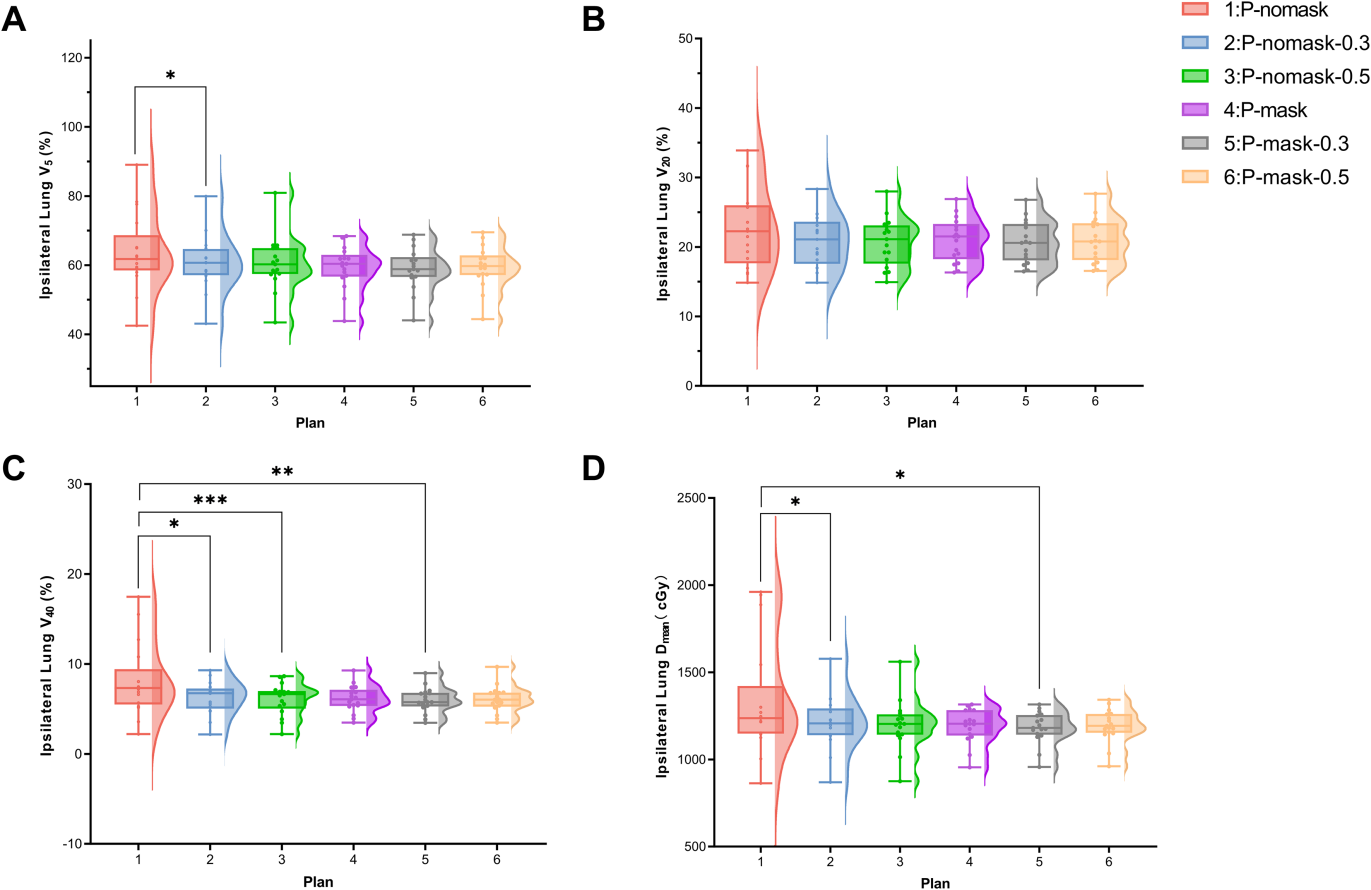
Dosimetric parameters of lung. **(A)** Ipsilateral Lung V_5_ (%). **(B)** Ipsilateral Lung V20 (%). **(C)** Ipsilateral Lung V_40_ (%). **(D)** Ipsilateral Lung D_mean_ (cGy). * p < 0.05, ** p < 0.01, *** p < 0.001 (Bonferroni-adjustedWilcoxon signed-rank test following Friedman test).

Ipsilateral lung V5 was generally similar across groups, though Plan 1 had significantly higher V5 than Plan 2 (p < 0.05), suggesting greater low-dose exposure without mask or bolus (**Figure 3A**). No significant differences were observed for V20 (p > 0.05), but bolus showed a trend toward dose reduction (**Figure 3B**). For V40, Plan 1 was significantly higher than Plans 2 (p < 0.05) and 3 (p < 0.001), indicating bolus reduces high-dose lung exposure (**Figure 3C**). Dmean was higher in Plan 1 than in Plans 2 (p < 0.05) and 5 (p < 0.05), showing bolus and mask use help lower lung dose (**Figure 3D**). Overall, non-mask, non-bolus setups increased lung exposure, while bolus effectively reduced lung dose, particularly in high-dose regions.

#### 3.3.2 Cardiac dosimetry

Cardiac dose changes were minimal and nonsignificant (P > 0.05) (**Supplementary Figure S1**).

### 3.4 Radiation delivery efficiency

**Figure 4:**
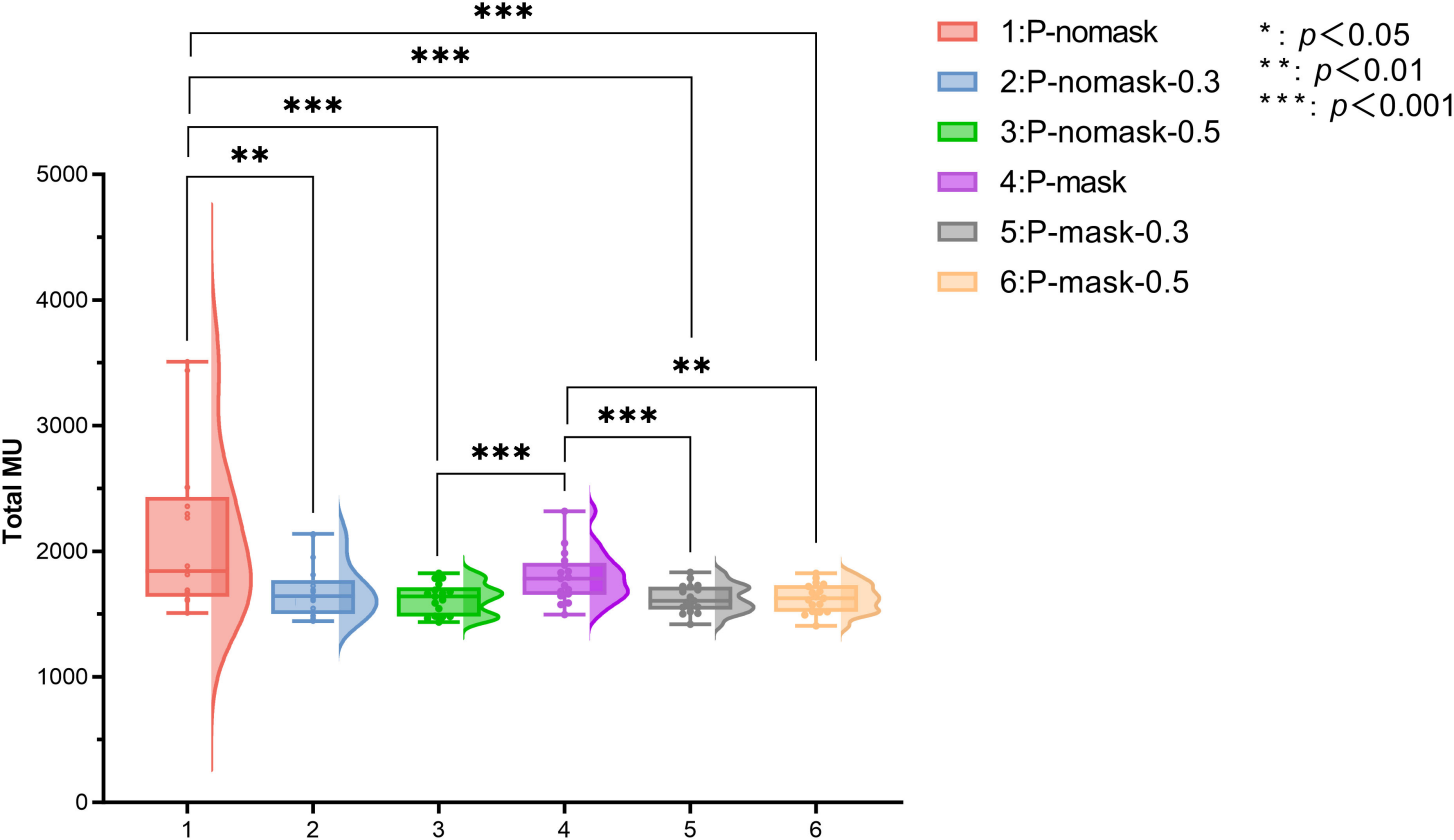
Radiation delivery efficiency. * p < 0.05, ** p < 0.01, *** p < 0.001 (Bonferroni-adjusted Wilcoxon signed-rank test following Friedman test).

A significant difference in output dose was observed among the six plans (p < 0.001). Plan 1 had a median MU of 1782.0 (IQR: 1653.0–1907.5). In the P-nomask group, bolus plans (2 and 3) showed significantly lower output doses than Plan 1 (p < 0.01), with no difference between bolus thicknesses (0.3 *vs*. 0.5cm; p > 0.05). Comparison of Plan 1 and Plan 4 showed no significant difference (p > 0.05), indicating that mask use alone does not affect efficiency. In the P-mask group, adding bolus (Plans 5 and 6) further reduced output dose (p < 0.01), while thickness differences remained nonsignificant (p > 0.05). Overall, bolus significantly reduces output dose, with minimal effect of thickness, and masks alone have no independent impact but enhance dose reduction when combined with bolus (**Figure 4**).

### 3.5 Relationship between chest wall thickness and dosimetric parameters in the target area

Patients were stratified by average chest wall thickness into low (<4.1cm), intermediate (4.1–5.0cm), and high (>5.0cm) groups. Six radiotherapy plans (P-mask, P-mask-0.3, P-mask-0.5, P-nomask, P-nomask-0.3, P-nomask-0.5) were compared within each group (**Tables 3**–**5**; **Supplementary Figures S2**-**S4**), and correlations between chest wall thickness and target dosimetric parameters are presented in **Supplementary Figure S5**.

**Table 3:** Comparison of the dosimetric parameters in the low chest wall thickness group of patients.

Parameters	P-mask	P-mask-0.3	P-mask-0.5	P-nomask	P-nomask-0.3	P-nomask-0.5	χ^2^	*P*
Skin								
D_min_ at 3mm Depth (cGy)	2959.35(2655.08,3326.98)	2946.90(2820.15,3627.73) d	3066.00(2782.725,3380.400)	1473.20(1129.63,1664.43)	2832.60(1604.18,3219.40)	2770.45(1574.80,3254.00)	17.71	**
D_min_ at 5mm Depth (cGy)	2895.70(2612.75,3284.32)	2944.75(2690.45,3626.95) df	3085.05(2640.28,3379.98) d	1482.55(1336.93,1745.48)	2802.80(1630.85,3163.98)	2464.45(1599.48,2839.30)	20.76	**
D_max_ at 3mm Depth (cGy)	5933.70(5839.13,6091.13)	5869.950(5795.93,6062.83)	5879.150(5809.83,6088.73)	5870.75(5705.45,7738.38)	5821.00(5749.85,5914.85)	5870.75(5753.00,5965.50)	5.24	ns
D_max_ at 5mm Depth (cGy)	5962.65(5849.40,6097.15)	5869.95(5809.25,6102.55)	5892.85(5815.53,6113.35)	5949.65(5849.05,7738.38)	5826.00(5797.28,5952.58)	5882.15(5776.03,6181.35)	10.57	ns
D_mean_ at 3mm Depth (cGy)	5281.30(521.48,5386.23)	5314.45(5281.63,5400.45)	5320.80(5279.78,5464.98) de	4428.650(4171.08,5552.18)	5101.950(5049.93,5245.80)	5165.000(5129.30,5271.38)	18.29	**
D_mean_ at 5mm Depth (cGy)	5316.90(5267.53,5449.73)	5328.35(5289.93,5418.0)	5327.30(5286.58,5465.68)	4858.00(4620.15,5854.40)	5180.85(5134.43,5286.03)	5202.50(5183.18,5277.85)	17.14	**
Skin ≥V_105%_ at 3mm (%)	52.62 (41.37,71.08)	62.21(52.14,65.914)	62.464(52.414,69.334) e	29.484(8.53,60.56)	35.90 (28.65,60.12)	43.82 (38.41,63.75)	14.10	*
Skin ≥V_105%_ at 5mm (%)	62.08 (54.17,74.45)	64.35 (59.46,70.39) e	64.02 (58.71,72.30)	38.07(30.50,76.58)	45.37 (40.75,64.79)	49.08 (43.99,63.91)	16.57	**
Skin ≥V_110%_ at 3mm (%)	22.52 (12.15,34.85)	25.90 (20.34,34.19) e	26.48(21.60,37.36)	2.27(0.71,50.75)	11.16 (6.61,21.60)	18.30 (10.76,29.24)	14.10	*
Skin ≥V_110%_ at 5mm (%)	29.40 (22.66,37.21)	28.71(22.33,36.19)	28.56(21.89,38.07)	15.31 (9.77,68.07)	16.47(11.97,27.01)	19.83 (13.99,28.35)	9.33	ns
Target Volume								
CI	0.83 (0.78,0.85)	0.80 (0.77,0.83)	0.78 (0.74,0.82)	0.84(0.64,0.85)	0.82 (0.81,0.84)	0.811 (0.79,0.83)	11.60	*
HI	0.19(0.17,0.20) f	0.18(0.16,0.19)	0.18(0.16,0.19)	0.20(0.17,0.56)	0.16(0.15,0.19)	0.16(0.15,0.17)	15.05	*
V_95%_ (%)	98.01(97.58,98.40)	98.34(98.16,98.50)	98.32(98.14,98.51) f	97.12(95.90,98.50)	98.51(98.21,98.76)	98.61 (98.45,98.77)	12.48	*
D_max_(cGy)	6003.80(5698.90,6133.05)	5840.05(5402.475,6128.08)	5900.95(5672.15,6134.40) f	5969.45(5889.53,7738.38)	5885.7(5836.80,6023.90)	5860.25(5332.88,5971.40)	13.43	*
OAR								
Ipsilateral Lung V_5_ (%)	61.15(57.61,68.11)	59.64(57.30,67.76)	60.19(58.00,68.35)	63.80(59.17,81.03)	62.76 (58.44,72.54)	62.93(58.66,69.53)	8.67	ns
Ipsilateral Lung V_20_ (%)	22.00(18.65,23.95)	21.69(18.55,24.30)	21.89(18.70,24.57)	21.43 (19.09,32.24)	20.79 (18.77,25.62)	20.85 (18.95,24.44)	8.48	ns
Ipsilateral Lung V_40_ (%)	6.16 (5.11,7.40)	6.06 (5.14,7.28)	6.10 (5.16,7.51)	7.33(6.58,13.41) f	6.76(5.36,7.65)	6.74 (5.11,7.47)	13.05	*
Ipsilateral Lung D_mean_ (cGy)	1223.60(1167.35,1310.58)	1204.40(1166.50,1299.53)	1214.90(1174.83,1326.90)	1241.80(1200.23,1901.65)	1216.7(1171.68,1404.03)	1219.90(1174.85,1328.85)	11.32	*
Efficiency								
Output dose (MU)	1835(1686.25,1959.50) f	1614.50(1554.75,1797.00)	1632.00(1577.75,1797.50)	2040.50(1645.25,2646.50) f	1684.00(1531.50,2131.75)	1663.50(1528.00,1715.00)	19.93	**

* p < 0.05, ** p < 0.01 (Bonferroni-adjusted Wilcoxon signed-rank test following Friedman test). ns, not statistically significant.

**Table 4:** Comparison of the dosimetric parameters in the intermediate chest wall thickness group of patients.

Parameters	P-mask	P-mask-0.3	P-mask-0.5	P-nomask	P-nomask-0.3	P-nomask-0.5	χ^2^	*P*
Skin								
D_min_ at 3mm Depth (cGy)	3118.70(2985.38,3129.60) df	3063.30(2922.45,3117.95)	3023.45(2847.20,3 123.03)	1474.70(1206.70,1823.65)	1792.30(1546.28,2016.75)	1736.75(1543.43,1846.00)	26.10	***
D_min_ at 5mm Depth (cGy)	3053.35(2927.88,3127.58) f	3045.45(2922.45,3150.88) df	3003.05(2811.50)	1455.00(1318.55,1717.23)	1675.95(1478.88,1941.05)	1644.75(1476.30,1804.95)	23.91	***
D_max_ at 3mm Depth (cGy)	5901.85(5817.40,6008.80)	5779.35(5772.63,5838.28) d	5781.15(5768.20,5847.00) d	6417.35(5945.65,7183.40)	5798.60(5730.10,5910.05)	5820.6(5745.55,5876.68)	18.00	**
D_max_ at 5mm Depth (cGy)	5902.80(5828.10,6034.98) c	5801.40(5783.43,5846.30) d	5789.35(5775.70,5862.25) d	6417.35(5945.65,7200.53)	5843.90(5791.10,5956.48)	5842.55(5784.70,5886.50) d	19.62	*
D_mean_ at 3mm Depth (cGy)	5214.65(5074.08,5339.43)	5218.30(5181.28,5299.43)	5220.15(5174.30,5305.13)	4860.60(4230.70,5404.93)	5184.15(4999.08,5244.78)	5230.90(5052.53,5267.63)	11.81	*
D_mean_ at 5mm Depth (cGy)	5258.60(5155.98,5385.50)	5251.60(5211.70,5336.08)	5248.05(5201.65,5333.23)	5081.60(4669.98,5744.48)	5224.40(5092.35,5295.30)	5245.60(5117.70,5262.20)	7.49	ns
Skin ≥V_105%_ at 3mm (%)	51.46(34.10,69.85)	49.97(44.51,63.34)	48.80(43.32,64.02)	39.08 (5.41,59.00)	50.48(26.27,58.38)	58.87(33.79,59.67)	4.00	ns
Skin ≥V_105%_ at 5mm (%)	58.45(45.68,75.63)	55.01 (50.09,69.42)	53.89(48.25,68.85)	57.01(33.33,73.75)	58.17 (36.93,63.82)	59.35(40.25,61.32)	5.52	ns
Skin ≥V_110%_ at 3mm (%)	21.34 (6.76,40.01)	19.36 (12.28,30.03)	19.33 (12.33,31.68)	20.13 (0.91,50.00)	15.69 (3.47,21.28)	23.95(7.41,26.68)	6.29	ns
Skin ≥V_110%_ at 5mm (%)	25.36(14.90,41.62)	21.94(15.36,32.90)	21.10(14.79,31.57)	32.15 (7.27,67.01)	22.40(10.10,26.99)	24.90(12.51,27.69)	5.43	ns
Target Volume								
CI	0.85(0.83,0.88) c	0.83(0.81,0.84)	0.81 (0.80,.83)	0.83(0.67,0.87)	0.83 (0.82,0.85)	0.83(0.802,0.85)	16.76	**
HI	0.17 (0.16,0.23)	0.16 (0.15,0.17)	0.16(0.13,0.17) d	0.32 (0.17,0.55)	0.17(0.16,0.19)	0.16(0.15,0.18) d	18.57	**
V_95%_ (%)	98.44 (97.23,98.71)	98.72 (98.46,98.96)	98.72 (98.46,98.92) f	96.66 (94.89,98.44)	98.49 (98.31,98.78)	98.64(98.37,99.04)	19.71	**
D_max_(cGy)	98.44 (97.23,98.71)	98.72 (98.46,98.96) d	98.72 (98.46,98.96) d	6417.35(5883.43,7200.53)	5889.95(5877.00,6019.95)	5888.85(5862.43,5965.98)	18.47	**
OAR								
Ipsilateral Lung V_5_ (%)	60.25 (57.73,62.77)	59.01(57.07,62.45)	59.65 (57.41,62.96)	62.75 (58.85,73.57)	59.69(56.58,64.87)	58.90 (57.07,65.12)	8.48	ns
Ipsilateral Lung V_20_ (%)	21.66(19.80,23.82)	21.15(19.61,23.32)	21.25 (19.73,23.44)	22.92(20.77,27.63)	22.15 (18.88,23.37)	22.10 (19.28,23.09)	4.95	ns
Ipsilateral Lung V_40_ (%)	6.89 (5.37,7.55)	6.44 (5.52,7.22)	6.43(5.55,7.18)	7.71 (6.28,12.46)	7.08 (5.65,8.11)	6.69 (5.73,8.07)	9.62	ns
Ipsilateral Lung D_mean_ (cGy)	1211.15(1181.20,1282.33)	1206.80(1160.95,1253.65)	1211.90(1167.63,1259.65)	1253.15(1208.03,1647.98)	1218.65(1135.95,1321.18)	1218.90(1146.95,1284.50)	9.43	ns
Efficiency								
Output dose (MU)	1760.00(1555.75,1996.90)	1641.50(1484.50,1699.52) d	1641.25(1489.50,1694.50) d	2403.00(1814.25,2741.50)	1656.65(1504.25,1782.50)	1639.35(1476.00,1750.50) d	18.48	**

* p < 0.05, ** p < 0.01, *** p < 0.001 (Bonferroni-adjusted Wilcoxon signed-rank test following Friedman test). ns, not statistically significant.

**Table 5:** Comparison of the dosimetric parameters in the high chest wall thickness group of patients.

Parameters	P-mask	P-mask-0.3	P-mask-0.5	P-nomask	P-nomask-0.3	P-nomask-0.5	χ^2^	*P*
Skin								
D_min_ at 3mm Depth (cGy)	3077.90(2464.85,3296.05)	2997.20(2584.90,3668.20) d	3005.00(2599.90,3683.40) d	1099.40(1073.65,1350.65)	1940.60(1634.3,3162.50)	1940.80(1628.60,3335.35)	14.60	
D_min_ at 5mm Depth (cGy)	3085.10(2451.15,3231.35)	3089.80(2474.85,3576.20) d	2927.70(2260.10,3400.85) d	1260.10(1188.40,1364.65)	1867.90(1543.15,3074.70)	1868.60(1537.05,3213.45)	13.23	*
D_max_ at 3mm Depth (cGy)	5819.40(5773.15,6046.20) e	5846.50(5755.80,5990.95)	5875.80(5767.40,6018.80)	5767.00(5601.25,5892.00)	5737.80(5690.10,5896.90)	5758.90(5729.90,5910.45)	13.00	*
D_max_ at 5mm Depth (cGy)	5892.50(5794.35,6052.15)	5863.60(5773.65,6007.90)	5875.80(5782.10,6028.65)	5823.20(5767.50,5989.95)	5785.2(5751.7,5911.05)	5793.20(5753.15,5935.75)	8.77	ns
D_mean_ at 3mm Depth (cGy)	5158.90(4997.30,5267.75)	5203.90(5132.70,5267.90) d	5202.30(5164.85,5329.85) de	4111.30(3883.15,4205.85)	4955.70(4861.95,5026.40)	5007.90(4941.80,5124.15)	22.37	***
D_mean_ at 5mm Depth (cGy)	5210.50(5121.45,5281.40)	5230.80(5201.85,5317.95) d	5237.30(5222.15,5314.95) de	4561.80(4342.15,4638.00)	5041.30(4951,5120.20)	5072.80(5001.65,5175.45)	23.86	***
Skin ≥V_105%_ at 3mm (%)	43.68(25.76,57.85)	46.90 (37.61,57.50) d	46.00(41.00,57.86) d	4.35(2.49,9.66)	20.32 (18.19,31.38)	25.92 (23.54,40.63)	21.12	*
Skin ≥V_105%_ at 5mm (%)	50.40(40.81,61.04)	50.51(46.48,64.03) de	49.54(49.35,62.82) d	18.42(16.62,30.57)	30.42 (26.87,41.88)	33.09(29.72,46.48)	23.17	***
Skin ≥V_110%_ at 3mm (%)	17.41 (4.76,28.81)	19.34(10.63,27.15) d	19.58(13.36,28.03) de	0.55 (0.08,3.13)	3.99(2.36,11.04)	7.27(4.74,17.91)	21.46	*
Skin ≥V_110%_ at 5mm (%)	22.53(12.73,30.91)	22.22 (15.63,31.16)	22.15(17.94,30.79) d	5.33 (4.56,13.51) d	9.10 (7.57,16.91) d	10.75(8.57,20.85)	22.26	***
Target Volume								
CI	0.86 (0.84,0.88) cf	0.84 (0.82,0.86)	0.83 (0.80,0.85) d	0.86 (0.84,0.87)	0.84 (0.82,0.85)	0.83 (0.81,0.84) d	23.17	***
HI	0.16 (0.12,0.19)	0.16 (0.15,0.17)	0.16 (0.11,0.18)	0.16 (0.15,0.19)	0.16 (0.51,0.18)	0.15 (0.15,0.18)	5.46	ns
V_95%_ (%)	98.60 (98.33,98.96)	98.69 (98.50,99.05)	98.71 (98.50,99.02)	98.67 (98.40,99.02)	98.91 (98.54,99.06)	98.86 (98.55,99.05)	6.26	ns
D_max_(cGy)	5892.50(5810.20,6065.65)	5863.60(5810.20,6065.65)	5875.80(5815,6003.00)	5842.20(5810.75,6003.55)	5831.00(5819.10,5951.40)	5821.60(5800.40,5947.10)	9.91	ns
OAR								
Ipsilateral Lung V_5_ (%)	56.46(47.09,62.91)	56.48(47.35,62.34)	57.00 (47.86,62.83)	57.96 (46.53,62.25)	57.51(47.29,61.43)	57.87 (47.67,61.99)	3.74	ns
Ipsilateral Lung V_20_ (%)	18.83(17.08,22.38)	18.49 (17.08,22.15)	18.48(17.17,22.46)	16.95(15.49,23.59)	16.98 (15.57,22.93)	17.00(15.61,22.97)	5.69	ns
Ipsilateral Lung V_40_ (%)	5.44(4.11,6.34)	5.18(4.07,6.25)	5.15 (4.09,6.41)	5.17(2.89,7.33)	4.80 (2.83,6.93)	4.74(2.83,6.90)	5.11	ns
Ipsilateral Lung D_mean_ (cGy)	1130.20(990.35,1242.55)	1129.10(991.80,1225.35)	1150.60(997.80,1230.45)	1145.30(933.35,1259.35)	1136.10(940.30,1234.85)	1139.20(944.20,1239.35)	6.49	ns
Efficiency								
Output dose (MU)	1680.00(1617.00,1883.00) f	1563.00(1509.00,1722.50)	1520.00(1506.50,1735.50)	1670.00(1563.00,1766.00)	1608.00(1465.00,1728.00)	1614.00(1460.50,1712.50)	15.40	**

* p < 0.05, ** p < 0.01, *** p < 0.001 (Bonferroni-adjusted Wilcoxon signed-rank test following Friedman test). ns, not statistically significant.

#### 3.5.1 Skin dosimetric parameters

In the low-thickness group, most skin parameters (Dmin, Dmean at 3mm and 5mm; V105% and V110% at 3mm) differed significantly among the six plans (p < 0.05), while Dmax and V110% at 5mm showed no significant change, suggesting limited sensitivity to compensation (**Table 3**, **Supplementary Figure S2**). In the intermediate group, only Dmax and Dmean at 3mm differed significantly (p < 0.05), indicating a modest bolus effect (**Table 4**, **Supplementary Figure S3**). In the high-thickness group, P-mask with bolus plans had higher Dmean and V105%/V110%, but inter-plan differences were smaller; some parameters (Dmin, Dmean at both depths) remained significant (p < 0.05), while Dmax and V110% at 5mm did not (**Table 5**, **Supplementary Figure S4**).

#### 3.5.2 Target volume dosimetric parameters

CI, HI, V95%, and Dmax varied across chest wall groups. In the low-thickness group, all indices showed significant inter-plan differences (χ² = 11.60–15.05, p < 0.05), with bolus plans, especially P-mask-0.5 and P-nomask-0.5, improving HI and V95%, indicating better homogeneity and coverage (**Table 3**). In the intermediate group, all indices remained significant (p < 0.05), confirming bolus efficacy (**Table 4**). In the high-thickness group, HI and V95% differences were no longer significant (p > 0.05), though CI remained sensitive (χ² = 23.17, p < 0.001, **Table 5**), suggesting bolus-induced improvements diminish with thicker chest walls.

#### 3.5.3 Ipsilateral lung dosimetric parameters

In the low-thickness group, V40 and Dmean differed significantly among plans (χ² = 13.05 and 11.32, p < 0.05), with higher doses in non-bolus plans, while V5 and V20 showed no significant differences (**Table 3**, **Supplementary Figure S2**). In the intermediate group, no lung parameters reached significance, though non-bolus plans trended toward higher Dmean (**Table 4**, **Supplementary Figure S3**). In the high-thickness group, all lung dose indices were comparable across plans (p > 0.05) and overall exposure was lower than in thinner chest walls (**Table 5**, **Supplementary Figure S4**).

#### 3.5.4 Radiation delivery

Output dose, reflecting radiation delivery efficiency, were also compared across the three chest wall thickness groups. Output dose differed significantly among plans in all thickness groups (low: χ² = 19.93; intermediate: χ² = 18.48; high: χ² = 15.40; p < 0.01). In low- and intermediate-thickness groups, P-nomask plans had the highest output dose, while bolus plans—particularly P-mask-0.3 and P-mask-0.5—showed lower doses, indicating improved treatment efficiency (**Tables 3**, **4**). In the high-thickness group, differences were smaller, with P-mask-0.5 yielding the lowest output dose (**Table 5**). Across all groups, bolus consistently reduced output dose, with minimal difference between 0.3cm and 0.5cm thicknesses.

#### 3.5.5 Correlation analysis

As shown in **Supplementary Figure S5**, chest wall thickness demonstrated a negative correlation with skin and lung dose parameters. Specifically, increases in chest wall thickness were associated with decreases in skin D_mean_(r=-0.25, p<0.05 in 3mm; r=-0.26, p<0.01 in 5mm), V_105%_(r=-0.4, p<0.001 in 3mm; r=-0.43, p<0.001 in 5mm), and V_110%_(r=-0.29, p<0.01 in 3mm; r=-0.29, p<0.01 in 5mm), as well as reductions in ipsilateral lung V_20_(r=-0.4, p<0.001), V_40_(r=-0.38, p<0.001), D_mean_(r=-0.43, p<0,001) and MU(r=-0.24, p<0.05).

## 4 Discussion

This study investigated the effects of radiotherapy techniques—including thermoplastic mask use and varying bolus thickness—on target delineation and dose distribution in post-mastectomy breast cancer patients. Bolus application improved dose homogeneity and target coverage while reducing high-dose lung exposure and overall output dose, enhancing treatment efficiency, particularly in patients with thinner chest walls. In contrast, thermoplastic mask use alone increased skin dose, indicating a compensatory effect independent of bolus.

We observed a negative correlation between chest wall thickness and both skin and lung dosimetric parameters: thicker chest walls were associated with lower skin Dmean, V_105%_, V_110%_, and reduced ipsilateral lung V_20_, V_40_, Dmean, and output dose, reflecting better natural dose attenuation. Most heart dosimetric parameters showed no significant differences, likely due to the limited sample size, especially for left-sided cases.

Bolus application improved target dose distribution and reduced high-dose lung exposure but increased skin dose. For patients with skin invasion or recurrence, bolus is recommended to enhance dermal dose, but potential acute skin toxicity should be considered (15, 16). While thermoplastic masks are essential for immobilization, they also increased skin dose, consistent with Lv et al. (9). Bolus thickness had greater impact on skin and OAR doses in non-mask patients, whereas masks provided partial compensation and reduced reliance on bolus adjustments. These findings support tailoring bolus strategies to the immobilization method, enabling flexible bolus selection in masked patients while preserving dosimetric quality, treatment efficiency, and comfort.

Our study confirms the dosimetric benefits of bolus use and identifies chest wall thickness as a key factor in postmastectomy radiotherapy planning. Subgroup analysis (<4.1 cm, 4.1–5.0 cm, >5.0 cm) revealed that thinner chest wall patients showed greater variability in skin dose, target coverage, and lung exposure. Bolus improved target homogeneity, coverage, and treatment efficiency in low and intermediate chest wall groups but had minimal impact in thicker chest walls. Chest wall thickness was negatively correlated with skin and lung doses, indicating natural dose attenuation. Overall, bolus use is most advantageous in thinner chest wall patients, whereas compensation in thicker chest walls can be individualized.

This study has several limitations. The small sample size and single-center design may limit generalizability, and subjective factors in target and OAR contouring could introduce systematic errors. Additionally, the chest wall thickness stratification requires validation in larger cohorts. Future studies should involve larger, multicenter populations to improve statistical power and external validity. Advanced image segmentation and automated contouring could reduce subjectivity, and multidimensional analyses integrating positional and anatomical data may better clarify the relationship between chest wall thickness and dosimetric parameters.

This study offers important insights for optimizing radiotherapy planning in post-mastectomy breast cancer. Our results show that the judicious selection of bolus material and immobilization methods can significantly influence target dose distribution and organ-at-risk protection. The inverse correlations between chest wall thickness and lung dose, skin dose distribution, as well as delivery efficiency, provide a promising basis for individualizing radiotherapy plans. Although the number of included patients was relatively small, each patient served as their own control, thereby minimizing inter-patient variability and enhancing the reliability of intra-patient dosimetric comparisons. As an exploratory dosimetric study, these findings provide valuable insights for optimizing bolus and mask application in PMRT, while larger multicenter cohorts are warranted to confirm their generalizability. Future research should focus on validating these findings and exploring adaptive planning strategies based on chest wall anatomy, ultimately paving the way for more personalized and safer radiotherapy protocols.

## Conclusion

Bolus and mask use significantly affect dosimetric outcomes in PMRT. Bolus improves homogeneity and efficiency but increases skin dose, particularly in thin-chest-wall patients. Chest wall thickness should guide bolus application to optimize PMRT planning.

## Data Availability

The raw data supporting the conclusions of this article will be made available by the authors, without undue reservation.

## Supplementary material

Supplementary Figure 1 Dosimetric Parameters of Cardiac. **(A)** Heart V_5_ (%)-Left. **(B)** Heart V_10_ (%)-Left. **(C)** Heart V_20_ (%)-Left. **(D)** Heart V_5_ (%)-Right. **(E)** Heart V_10_ (%)-Right. **(F)** Heart V_20_ (%)-Right. **(G)** Heart D_mean_-Left (cGy). **(H)** Heart D_mean_-Right(cGy).

Supplementary Figure 2 Dosimetric parameters in the low chest wall thickness group.

Supplementary Figure 3 Dosimetric parameters in the intermediate chest wall thickness group.

Supplementary Figure 4 Dosimetric parameters in the high chest wall thickness group.

Supplementary Figure 5 Correlation analysis of chest wall thickness with dosimetric parameters.
