# Supplementary figures and images for: Dosimetric impact of bolus thickness and immobilization mask use in postmastectomy radiotherapy: a chest wall thickness–based analysis
Source: Front Oncol. 2026 Jan 20;15:1716042. doi: 10.3389/fonc.2025.1716042 (PMC12866897; doi:10.3389/fonc.2025.1716042)

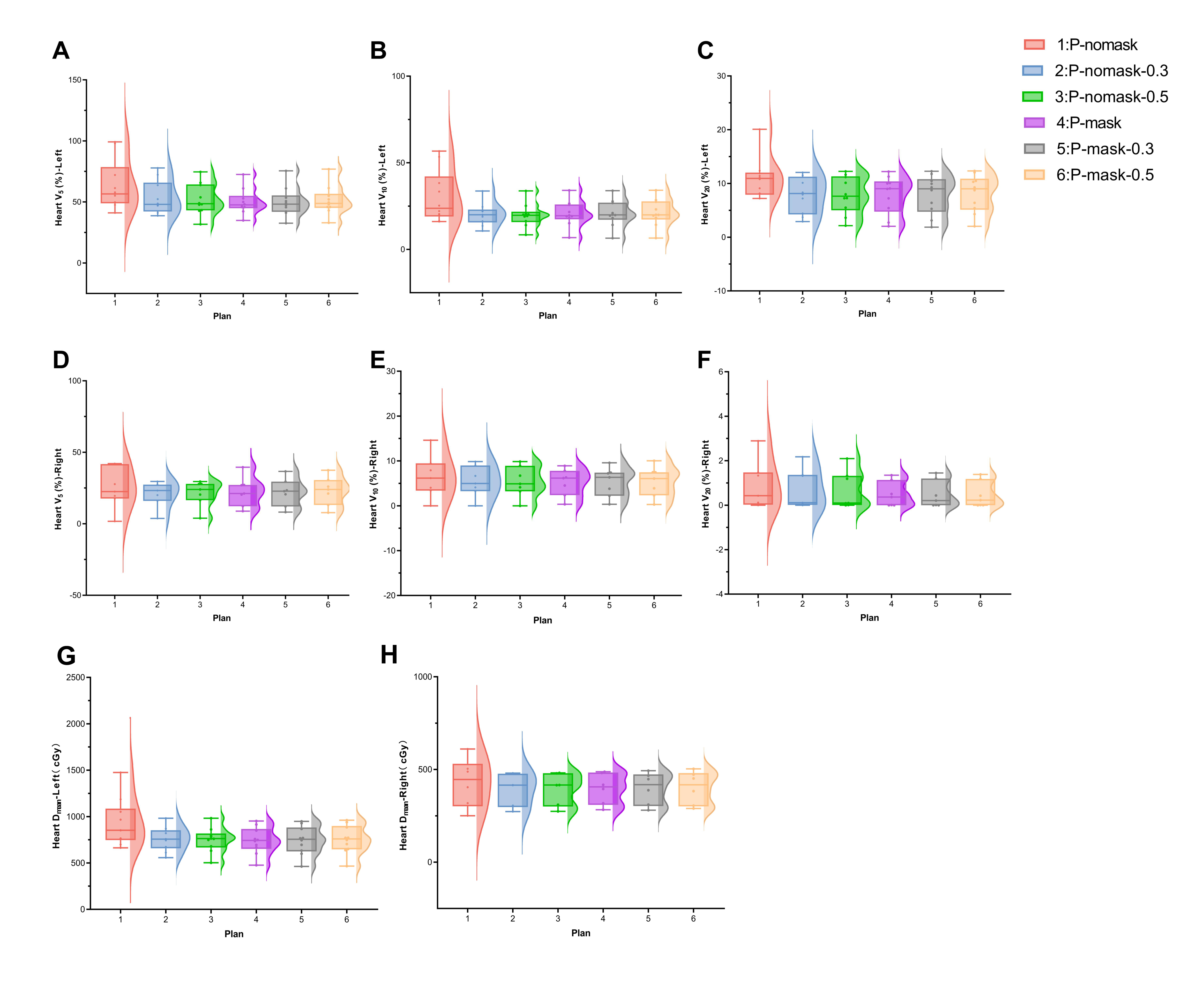

Supplement: Supplementary Figure 1 — Dosimetric Parameters of Cardiac. (A) Heart V5 (%)-Left. (B) Heart V10 (%)-Left. (C) Heart V20 (%)-Left. (D) Heart V5 (%)-Right. (E) Heart V10 (%)-Right. (F) Heart V20 (%)-Right. (G) Heart Dmean-Left (cGy). (H) Heart Dmean-Right(cGy). [file Image1.tif]

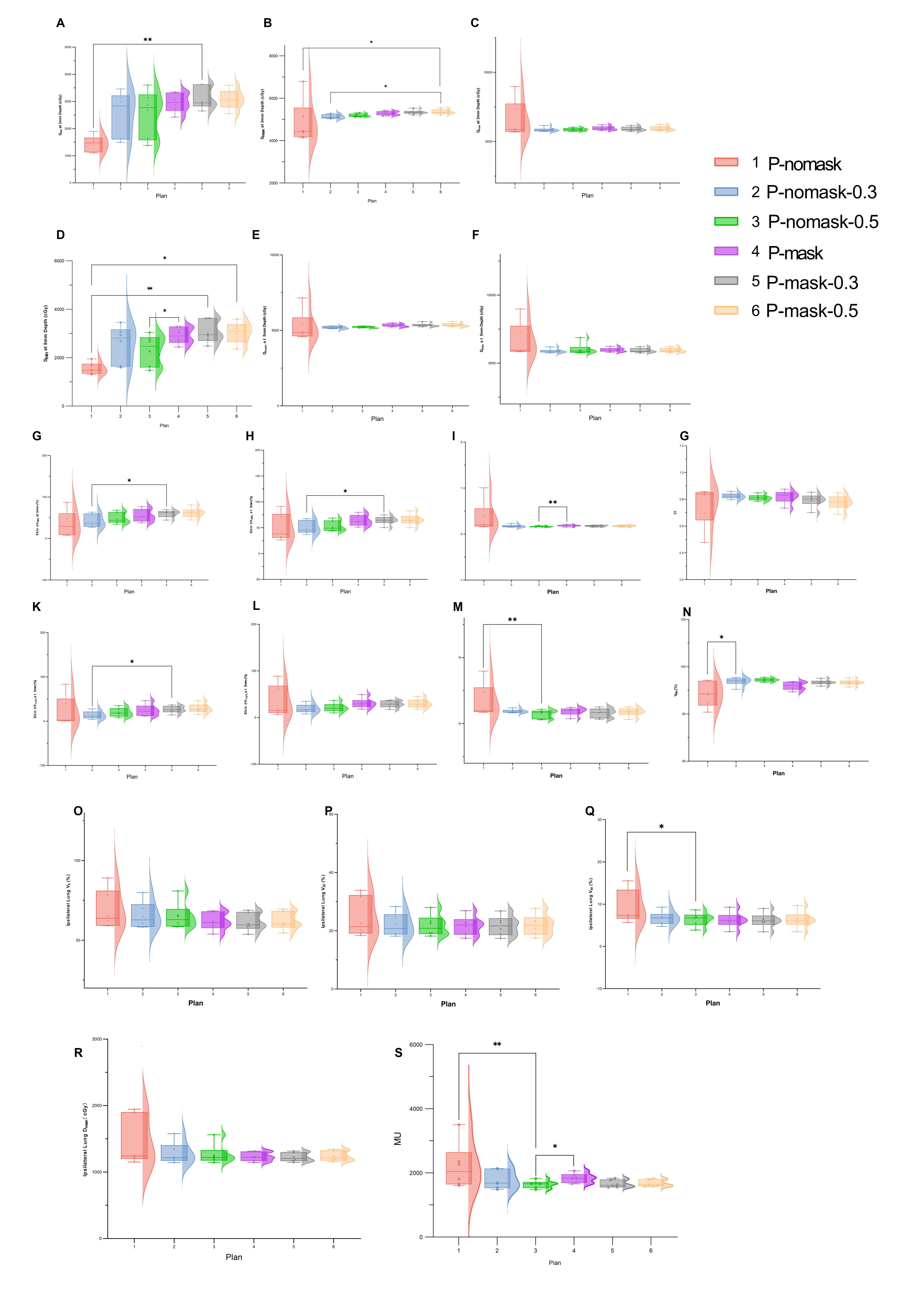

Supplement: Supplementary Figure 2 — Dosimetric parameters in the low chest wall thickness group. [file Image2.tif]

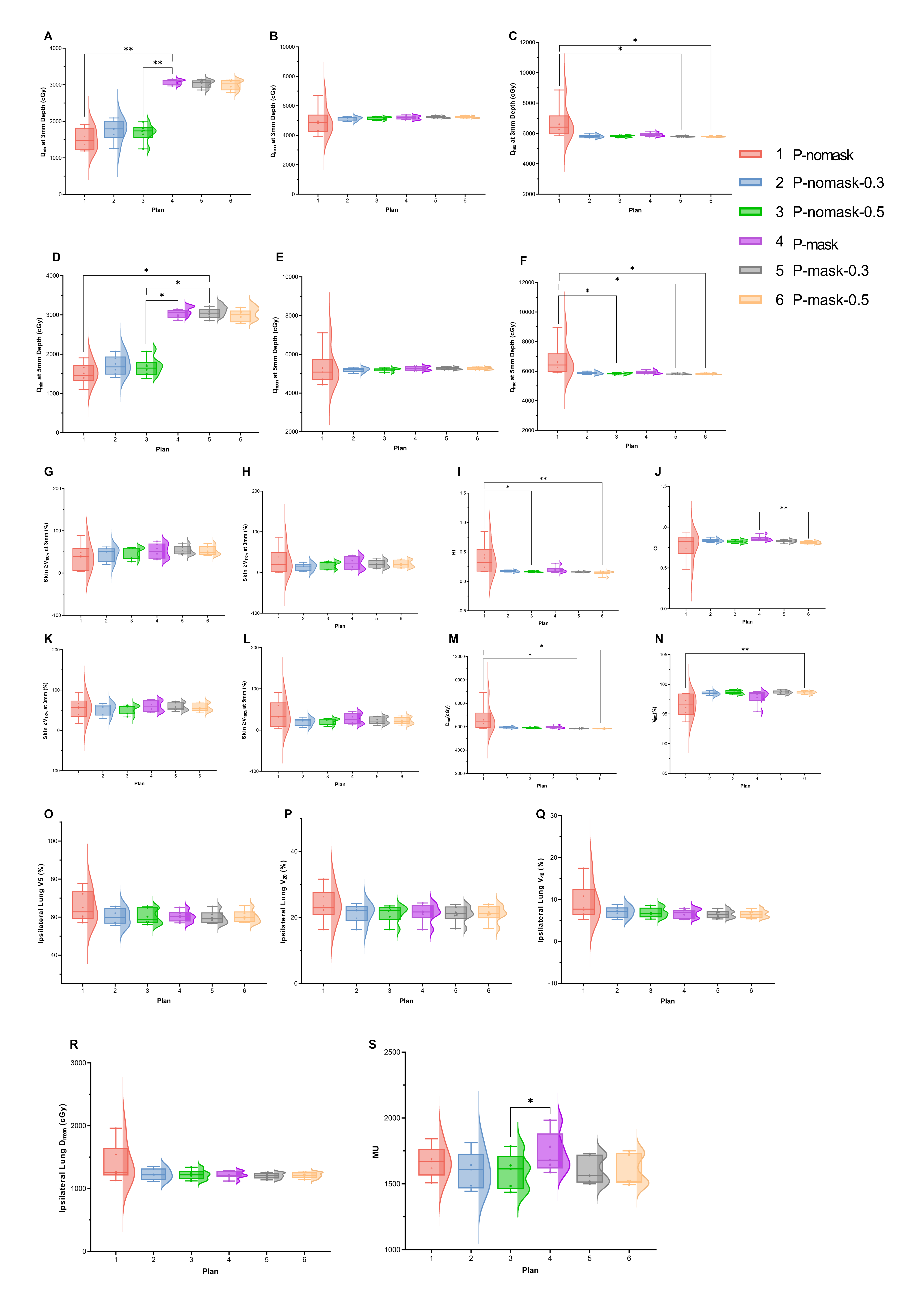

Supplement: Supplementary Figure 3 — Dosimetric parameters in the intermediate chest wall thickness group. [file Image3.tif]

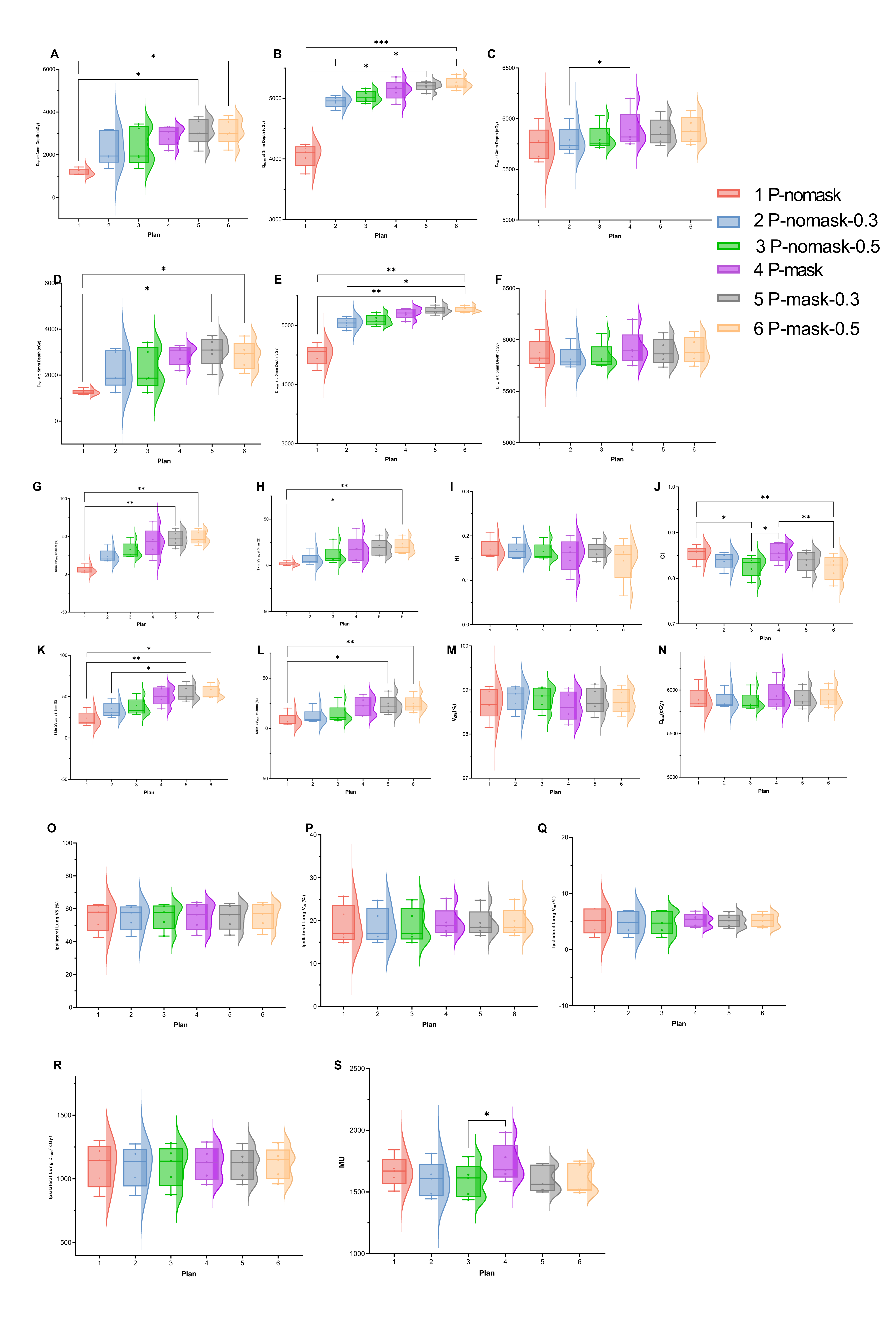

Supplement: Supplementary Figure 4 — Dosimetric parameters in the high chest wall thickness group. [file Image4.tif]

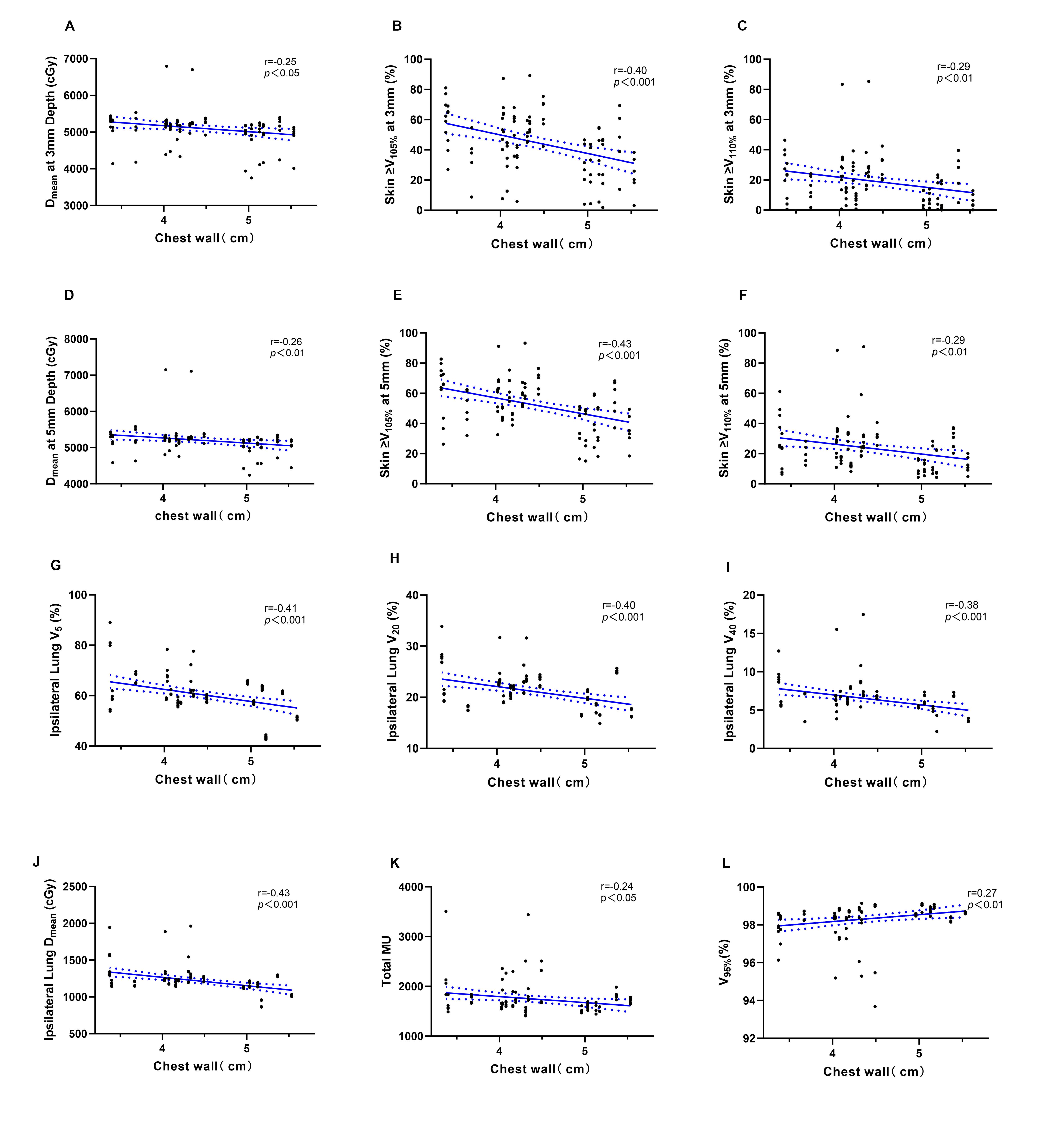

Supplement: Supplementary Figure 5 — Correlation analysis of chest wall thickness with dosimetric parameters. [file Image5.tif]
